# Vitronectin promotes immunothrombotic dysregulation in the venular microvasculature

**DOI:** 10.3389/fimmu.2023.1078005

**Published:** 2023-02-08

**Authors:** Bernd Uhl, Florian Haring, Julia Slotta-Huspenina, Joshua Luft, Vera Schneewind, Jonas Hildinger, Zhengquan Wu, Katja Steiger, Bojan Smiljanov, Aarif M. N. Batcha, Oliver T. Keppler, Johannes C. Hellmuth, Tobias Lahmer, Konrad Stock, Bernhard G. Weiss, Martin Canis, Konstantin Stark, Thomas Bromberger, Markus Moser, Christian Schulz, Wilko Weichert, Gabriele Zuchtriegel, Christoph A. Reichel

**Affiliations:** ^1^ Department of Otorhinolaryngology, University Hospital, Ludwig-Maximilians-Universität München (LMU), Munich, Germany; ^2^ Walter Brendel Centre of Experimental Medicine, University Hospital, Ludwig-Maximilians-Universität München (LMU) Munich, Munich, Germany; ^3^ Department of Pathology, Technical University of Munich, Munich, Germany; ^4^ Institute of Medical Data Processing, Biometrics, and Epidemiology (IBE), University Hospital, Ludwig-Maximilians-Universität München (LMU) Munich, Munich, Germany; ^5^ Data Integration for Future Medicine (DiFuture), University Hospital, Ludwig-Maximilians-Universität München (LMU) Munich, Munich, Germany; ^6^ Max von Pettenkofer Institute and Gene Center, Virology, National Reference Center for Retroviruses, Ludwig-Maximilians-Universität München (LMU) Munich, Munich, Germany; ^7^ German Centre for Infection Research (DZIF), Partner Site München, Munich, Germany; ^8^ Department of Medicine III, University Hospital, Ludwig-Maximilians-Universität München (LMU), Munich, Munich, Germany; ^9^ COVID-19 Registry of the LMU Munich (CORKUM), University Hospital, Ludwig-Maximilians-Universität München (LMU) Munich, Munich, Germany; ^10^ Department of Internal Medicine II, Technical University of Munich, Munich, Germany; ^11^ Department of Nephrology, Technical University of Munich, Munich, Germany; ^12^ Department of Cardiology, University Hospital, Ludwig-Maximilians-Universität München (LMU) Munich, Munich, Germany; ^13^ Institute of Experimental Hematology, Technical University of Munich, Munich, Germany

**Keywords:** immunothrombosis, platelets, neutrophils, microvasculature, systemic inflammation, SIRS, vitronectin

## Abstract

Microvascular immunothrombotic dysregulation is a critical process in the pathogenesis of severe systemic inflammatory diseases. The mechanisms controlling immunothrombosis in inflamed microvessels, however, remain poorly understood. Here, we report that under systemic inflammatory conditions the matricellular glycoproteinvitronectin (VN) establishes an intravascular scaffold, supporting interactions of aggregating platelets with immune cells and the venular endothelium. Blockade of the VN receptor glycoprotein (GP)IIb/IIIa interfered with this multicellular interplay and effectively prevented microvascular clot formation. In line with these experimental data, particularly VN was found to be enriched in the pulmonary microvasculature of patients with non-infectious (pancreatitis-associated) or infectious (coronavirus disease 2019 (COVID-19)-associated) severe systemic inflammatory responses. Targeting the VN-GPIIb/IIIa axis hence appears as a promising, already feasible strategy to counteract microvascular immunothrombotic dysregulation in systemic inflammatory pathologies.

## Introduction

Microvascular immunothrombotic dysregulation is a hallmark in the pathogenesis of severe non-infectious and infectious (*e.g.*, coronavirus disease 2019; COVID-19) systemic inflammatory conditions (1–5). Although the individual pathogenetic mechanisms vary widely between different inflammatory pathologies, platelets, immune cells, and plasmatic coagulation eventually collaborate for the formation of blood clots in the diseased microvasculature in a process termed immunothrombosis (1). Such thrombotic scaffolds usually support the recognition, containment, and elimination of pathogens in inflamed tissue, hence protecting the host’s integrity (1, 6, 7). Under severe inflammatory conditions, however, (microvascular) immunothrombosis is uncontrolled, which leads to thromboinflammation, ultimately resulting in the interruption of blood supply and subsequent ischemic organ dysfunction or failure (8–10). Whereas interventional procedures enable the revascularization of larger vessels in thrombotic disease, strategies for the dissolution of microvascular blood clots remain scarce due to their inaccessibility to these invasive techniques (11, 12).

The mechanisms underlying immunothrombosis substantially differ between different vascular beds. In lower-shear vessels (*e.g.*, larger arteries, veins, liver sinusoids), activated platelets stimulate neutrophils to release extracellular traps (NETs) (13–15). These networks of DNA and proteins, in turn, activate intrinsic and disinhibit extrinsic coagulation pathways to establish a fibrin matrix enforcing platelet clots (15–17). In contrast, thrombosis in the high shear microvasculature (vessel diameter < 100 µm) largely relies on rheological forces requiring specific molecular interactions between leukocytes, platelets, and endothelial cells. However, this process does not necessarily involve NET-dependent generation of fibrin frameworks (18–20) – most probably due to the limited stability of DNA-fibrin complexes under high shear (21). Instead, these cellular events depend on a complex molecular interplay between various molecular factors including von Willebrand factor (vWF), GPIbα, CD40, CD40L, thrombin, tissue factor (TF), neutrophil elastase, cathepsin G, and ATP (6, 22–24).

Vitronectin (VN) is a 75 kDa protein released by the liver and activated platelets. In the extracellular space, this multidomain glycoprotein is able to interact with a variety of proteins modulating different biological processes such as hemostasis (*via* PAI-1) and cell adhesion or migration (*via* αvβ3, αvβ5, αIIbβ3, and αvβ1 integrins) (25). Whereas VN has been demonstrated to stabilize thrombi in arteries (26, 27) and arterioles (28) by supporting platelet aggregation (29, 30), this protein has also been reported to exhibit anti-coagulant activity by inhibiting thrombin-fibrinogen interactions (31). The role of VN for clot formation in microvessels under inflammatory conditions, however, is still unclear. With respect to the ability of VN to interact with molecules expressed on platelets, immune cells, and/or microvascular endothelial cells as well as to regulate fibrinolysis, we hypothesize that this matricellular protein is critical for microvascular immunothrombosis in systemic inflammation.

## Materials and methods

### Ethics statement

All animal experiments were performed according to German law for animal protection and approved by the local government authorities (Regierung von „Oberbayern“). The use of human autopsy specimen was approved by the ethics committee of the TUM (Ref. 225/20S).

### Animals

Male C57BL/6J mice were purchased from Charles River (Sulzfeld, Germany). Male VN^-/-^ mice were generated as described previously (32) and backcrossed to the C57BL/6J background for 6 – 10 generations. All experiments were performed using mice at the age of 10-12 weeks. Animals were housed under conventional conditions with free access to food and water.

### Systemic inflammation model

Lipopolysaccharide (LPS, Escherichia coli O111:B4; 1 mg in 600 μl NaCl intraperitoneally (i.p.); Sigma-Aldrich, Schnelldorf, Germany) was used to induce a systemic inflammation driven by a robust release of pro-inflammatory cytokines ultimately leading to the development of systemic inflammatory response syndrome (SIRS) associated with subsequent organ failure such as acute respiratory distress syndrome (ARDS) (33, 34). After 6 hours, mice were sacrificed and flushed with PBS. Subsequently, organs were harvested.

### Confocal microscopy analyses of immunostained tissues and cells

For analyzing the formation of microvascular immunothrombi, liver, kidneys, and lungs of endotoxemic mice were embedded in TissueTek (Sakura Finetek) and frozen using dry ice. Afterwards, the organs were cut into 50 µm slices, put on glass slides, and fixed in 4% paraformaldehyde. Tissues were then washed with PBS and blocked with 2% Bovine Serum Albumin (BSA; Sigma Aldrich) in PBS for 60 minutes at 4° C. After another washing step with PBS, samples were incubated at 4° C for 24 hours with fluorescence-labeled monoclonal antibodies (mAb) directed against GPIbβ (Dylight649; emfret, Eibelstadt, Germany) and GR-1 (PE; Invitrogen, Waltham, Massachusetts, USA). In additional experiments, primary rabbit mAb directed against vitronectin (invitrogen), fibrin(ogen) (invitrogen), or fibronectin (invitrogen) were employed, followed by incubation with secondary AF-488-conjugated anti-rabbit antibodies for 120 minutes at room temperature. In further experiments, rabbit AF-555-conjugated anti-vWF (Bioss Inc., Woburn, Massachusetts, USA), rat Dylight649-conjugated anti-GPIbβ (emfret), and rat BV421-conjugated anti-GR-1 (biolegend, San Diego, California, USA) mABs were used. Immunostained tissues were mounted in PermaFluor (Beckman Coulter, Fullerton, CA). Z-stacks typically covering 30 μm (z-spacing 0.2 μm; objective magnification 20 x) were acquired using a Zeiss confocal laser-scanning microscope (LSM 900 with Airyscan 2; Carl Zeiss, Oberkochen, Deutschland). Acquired images were analyzed with Fiji ImageJ software (National Institutes of Health, Bethesda, Maryland, USA).

For the analysis of adhesion and signaling molecules in microvascular thrombi, excised mouse cremaster muscles were stained after induction of inflammation and photochemical injury as described below. Mouse cremaster muscles were then fixed in 4% paraformaldehyde. Subsequently, tissues were washed, blocked, and permeabilized in PBS, supplemented with 2% BSA (Sigma Aldrich) and 0.5% Triton X-100 (Sigma Aldrich) for 2 hours. After incubation at 4° C for 24 hours with rat mAb directed against GP1bβ (Dylight 488; emfret), GR-1 (brilliant violet 421; biolegend), CD11a (PE; biolegend), CD11b (PE; biolegend), CD54 (PE; biolegend), CD51 (PE; biolegend), or CD41/CD61 (PE, emfret), tissues were analyzed by confocal microscopy as described above.

### Flow cytometry analyses of platelets and immune cells

To study the effect of VN-deficiency on immune cell infiltration of liver, kidneys, and lungs of mice with severe systemic inflammation, organs were harvested 6 h after intraperitoneal injection of LPS (see above) and homogenized. The platelet count as well as the total white blood count of each organ homogenate was determined using a ProCyte Dx cell counter (IDEXX Laboratories, Westbrook, ME, USA). After incubation with anti-CD45-APC/Cy7 mAb (clone 30-F11; BD Bioscience), anti-CD11b-FITC mAb (clone M1/70; eBioscience, Waltham, Massachusetts, USA), anti-GR-1-PE mAb (clone RB6-8c5; eBioscience), anti-F4/80-eFluor 450 mAb (clone BM8; eBioscience) for 30 min on ice, lysis of erythrocytes was performed using lysing solution (BD Biosciences) for 10 min at room temperature. After washing, immunostained cells were analyzed on a flow cytometer (Gallios flow cytometer; Beckman Coulter Inc, Brea, California, USA). Approximately 20,000 gated events were collected in each analysis and isotype-matched controls were used in all experiments. Data analysis was performed using FlowJo software (Becton, Dickinson and Company). Absolute immune cell numbers were calculated using the white blood count and the relative share of the respective immune cell subset as assessed by flow cytometry.

### *In vivo* microscopy analyses of microvascular immunothrombosis in the mouse cremaster muscle

The surgical preparation of the mouse cremaster muscle was performed as originally described by Baez with minor modifications. Briefly, mice were anesthetized using a ketamine/xylazine mixture (100 mg/kg ketamine and 10 mg/kg xylazine), administered by i.p. injection. The left femoral artery was cannulated in a retrograde manner for administration of antibodies. The right cremaster muscle was exposed through a ventral incision of the scrotum. The muscle was opened ventrally in a relatively avascular zone, using careful electrocautery to stop any bleeding, and spread over the pedestal of a specialized custom-made microscopy stage. Epididymis and testicle were detached from the cremaster muscle and placed into the abdominal cavity. Throughout the procedure as well as after surgical preparation during *in vivo* microscopy, the muscle was superfused with warm buffered saline.

The setup for *in vivo* microscopy was centered around an AxioTech-Vario 100 Microscope (Zeiss MicroImaging GmbH, Goettingen, Germany), equipped with a Colibiri LED light source (Zeiss MicroImaging GmbH) for fluorescence epi-illumination microscopy. Light was directed onto the specimen *via* the filter set 62 HE (Zeiss MicroImaging GmbH) fitted with dichroic and emission filters [TFT 495 + 610 (HE); TBP 527 + LP615 (HE)]. Microscopy images were obtained with an AxioCam Hsm digital camera using a 20x water immersion lens (0.5 NA, Zeiss MicroImaging Gmbh). The images were processed with AxioVision 4.6 software (Zeiss MicroImaging GmbH).

Lipopolysaccharide (LPS, Escherichia coli O111:B4; 10 ng intrascrotally (i.s.); Sigma-Aldrich, Schnelldorf, Germany) was used to induce inflammation of the cremaster muscle. After 6 hours, microvascular thrombus formation was induced by photochemical injury as described before. Briefly, animals received an i.a. injection of a FITC-dextran solution (150 kDa, 2.5%, 6 ml/kg body weight, Sigma-Aldrich). Five minutes later, the vessel of interest was exposed to continuous epi-illumination using the FITC filter cube and appropriate illumination (λ = 488 nm). The field of illumination covered the entire field of view under investigation. To assure intergroup comparability, the mean fluorescence intensity was determined in each of the analyzed vessels immediately after onset of light exposure. The onset of the thrombus was defined as the first observation of endothelially adherent platelets during thrombus formation and the cessation of thrombus formation was defined as the complete occlusion of the vessel segment with flow cessation for over 60 s.

To determine the functional relevance of distinct adhesion and signaling molecules for microvascular thrombus formation in inflamed tissue, experiments were performed upon i.a. administration of blocking anti-CD11a mAbs (clone M17/4; 50 μg in 100 μl NaCl; biolegend, San Diego, CA, USA), anti-CD11b mAbs (clone M1/70; 50 μg in 100 μl NaCl; biolegend), anti-CD54 mAbs (clone YN1/1.7.4; 50 μg in 100 μl NaCl; biolegend), anti-CD51 mAbs (clone RMV-7; 50µg in 100µl NaCl; biolegend), or anti-CD61/41 mAbs (clone Leo.H4; 50 μg in 100 μl NaCl; emfret). In additional experiments, heparin (500 IU intraarterially (i.a.); ratiopharm GmbH, Ulm, Germany), a non-peptic GPIIb/IIIa inhibitor (GR144053 trihydrochloride; 10 mg/kg i.a.; 10 min prior to induction of thrombosis; R&D Systems, Lille, France), or drug vehicle were applied 10 min prior to induction of microvascular thrombus formation. Batroxobin (3 BU/kg; Defibrase^®^; DSM Nutritional Products Ltd, Branch Pentapharm, Switzerland) was applied i.p. 12 h prior to induction of microvascular thrombus formation.

To assess interactions of platelets, neutrophils, and endothelial cells during microvascular thrombogenesis, neutrophils were identified by anti-Ly6G mAbs (clone 1A8; 10 μg in 100 μl NaCl i.a.; BD Biosciences), platelets by anti-GPIbβ mABs (clone X-649; 10 μg in 100 μl NaCl i.a.; emfret), and endothelial cells by anti-PECAM-1/CD31 (clone 390; 3 μg in 100 μl NaCl i.a; eBiosciences).

### Rotational thromboelastometry

To analyze the effect of VN and vWF on hemostasis, recombinant mouse VN and vWF was added in different doses (1 ng/ml, 10 ng/ml, 100 ng/ml) to whole blood samples harvested from C57BL/6J mice. Rotational thromboelastometry (ROTEM, Pentapharm, Munich, Germany) was then performed (35). In separate experiments, the effect of a blocking anti-CD61/41 mAb upon stimulation with VN and vWF, of heparin or heparin together with a blocking anti-CD61/41 mAb upon stimulation with ADP, as well as of the effect of heteromerization of VN with recombinant murine PAI-1 (CPAI; 50 μg in 100 μl PBS; Molecular Innovations, Novi, MI) were evaluated.

### Platelet activation

To evaluate the effect of VN on platelet activation, platelets from the whole blood of C57BL/6J mice were incubated with PBS, recombinant mouse VN (100 ng/ml; sinobiological, Beijing, China), or ADP (10 ng/ml; Sigma-Aldrich, St. Louis, Missouri, USA). As a measure of platelet activation, surface binding of the high-affinity conformation-specific anti-integrin αIIbβ3 mAb conjugated with PE (clone JON/A; emfret) was assessed by flow cytometry (see above). Platelets were identified as CD45^-^ and CD41^+^ cells using anti-CD45 mAb labeled with APC/Cy7 (clone 30-F11; BD Bioscience) and anti-CD41 mAb conjugated with APC (clone MWReg30, biolegend). The cells were analyzed using the BD FACSCanto II (Becton, Dickinson and Company, Franklin Lakes, New Jersey, USA).

### Platelet spreading

Spreading of platelets from the whole blood of C57BL/6J mice was analyzed in blood smears mounted in PermaFluor by confocal microscopy upon incubation with PBS, recombinant murine VN (100 ng/ml; sinobiological, Beijing, China), or ADP (10 ng/ml; Sigma-Aldrich, St. Louis, Missouri, USA) for 60 minutes at 37°C on glass, collagen I, or fibrin(ogen). The spreading area of single platelets was measured using Fiji ImageJ.

### Platelet adhesion under flow

Platelet adhesion under varying shear rates was assessed in ibidi µ-slides VI0.1 (Ibidi, Gräfelfing, Germany). Slides were coated either with fibrin(ogen) (100 µg/ml in TBS, Sigma-Aldrich, Taufkirchen, Germany) or Vitronectin XF (25 µg/ml in TBS, STEMCELL Technologies, Köln, Germany) together with collagen-related peptide (CRP, 0.5 µg/ml, kindly provided by Prof. Siess, LMU, Munich) at 4°C overnight. Blood collected from wild-type mice was mixed with 1/2 volume of heparin (20 U/ml in TBS) and perfused through fibrinogen/CRP and vitronectin/CRP-coated flow chambers at defined flow rates resulting in 1, 5, 10 or 50 dyne/cm2 wall shear stress for 4 min using a PHD ULTRA pump (Harvard Apparatus, Holliston, MA, USA). Subsequently, flow chambers were washed by perfusing Tyrode’s buffer (136 mM NaCl, 0.43 mM NaH2PO4, 2.7 mM KCl, 12mM NaHCO3, 2 µM CaCl2, 1 µM MgCl2, 5 mM HEPES, 0.1% glucose, 0.35% BSA; pH 7.35) at the respective flow rate for 20 min and adherent platelets were stained with CellTrace CFSE dye (Thermo Fisher Scientific, Darmstadt, Germany). Images were acquired with an Evos M7000 life cell microscope (Thermo Fisher Scientific) and surface coverage was quantified using ImageJ software.

### Microvascular deposition of fibrin(ogen), vWF and VN in lungs of patients with severe systemic infectious inflammation (COVID-19), severe systemic non-infectious inflammation (pancreatitis-associated SIRS/ARDS), and of control patients

3 different groups with 10 individuals each were analyzed: i) lungs of patients with severe systemic infectious inflammation (COVID-19; individuals with COVID-19 confirmed by PCR for SARS-CoV-2 were included; autopsies were performed at the Institute of Pathology of the Technical University of Munich (TUM) between June 2020 and December 2020), ii) lungs of patients with severe systemic non-infectious inflammation (pancreatitis-associated SIRS/ARDS; individuals with clinically and histopathologically confirmed diagnosis were included; autopsies were performed at the Institute of Pathology of the Technical University of Munich (TUM) between 2001 and 2016), and iii) lungs of control patients (surgical tissue samples harvested from patients with a primary lung carcinoma; samples have been obtained from lung tissue far from the cancer region (> 3 cm) and have histopathologically been classified as “healthy/non-pathological”, particularly without signs of inflammation or infection; autopsies were performed at the Institute of Pathology of the Technical University of Munich (TUM) between 2008 and 2015).

In all individuals, an ultrasound-guided minimally invasive autopsy was performed, which included retrieval of biopsies (14-Gauge) of morphologically visible peripheral lung lesions or of morphologically healthy lung tissue. Biopsies were fixed in 4% formaldehyde for 72h, paraffin-embedded (FFPE) and 2µm slides were stained with hematoxylin & eosin (HE) and LADEWIG’s solution (12086.01000, Morphisto, Offenbach, Germany) according to standard procedures. IHC was performed using a vWF antibody (clone VWF/1859R, NSJ Bioreagents, V3491) diluted 1:100 in antibody diluent or a VN antibody (MA5-32157, Invitrogen) diluted 1:1000 in antibody diluent and stained on a Bond Rxm (Leica, Wetzlar, Germany) system after heat mediated antigen retrieval (ER1 for 30 min) using a Polymer Refine Detection Kit without post-primary antibody. Stained slides were scanned using an Aperio AT2 system (Leica, Wetzlar, Germany) and visualized using Aperio ImageScope (version 12.3.1.5011). Histopathological analyses of 2 different biopsy samples of 10 different individuals per group were analyzed in this study. For assessment of fibrin (bright red in LADEWIG staining), vWF (brown in immunostaining), and VN (brown in immunostaining) deposition in the microvasculature, four vessels with a diameter of >100 µm and four vessels with a diameter =< 100 µm were randomly selected for analysis in each biopsy sample. Presence of specific staining regardless of staining intensity was evaluated as positive.

### Statistics

Data analysis was performed with the statistical software SigmaPlot for Windows; Jandel Scientific, Erkrath, Germany). After confirming normality and equal variance of data (using the Shapiro-Wilk and Brown-Forsythe tests), the One-way ANOVA test followed by the Dunnett test (>2 groups) or the *t* test (2 groups) was used for the estimation of stochastic probability in intergroup comparisons. If normality and/or equal variance testing failed, the Kruskal-Wallis One-way ANOVA of Ranks test followed by the Dunnett test (>2 groups) or the Mann-Whitney rank sum test (2 groups) was used. For survival estimation, the log rank test was employed. Mean values and SEM are given. *P* values <0.05 were considered significant.

## Results

### VN promotes immunothrombosis in the venular microvasculature

**Figure 1:**
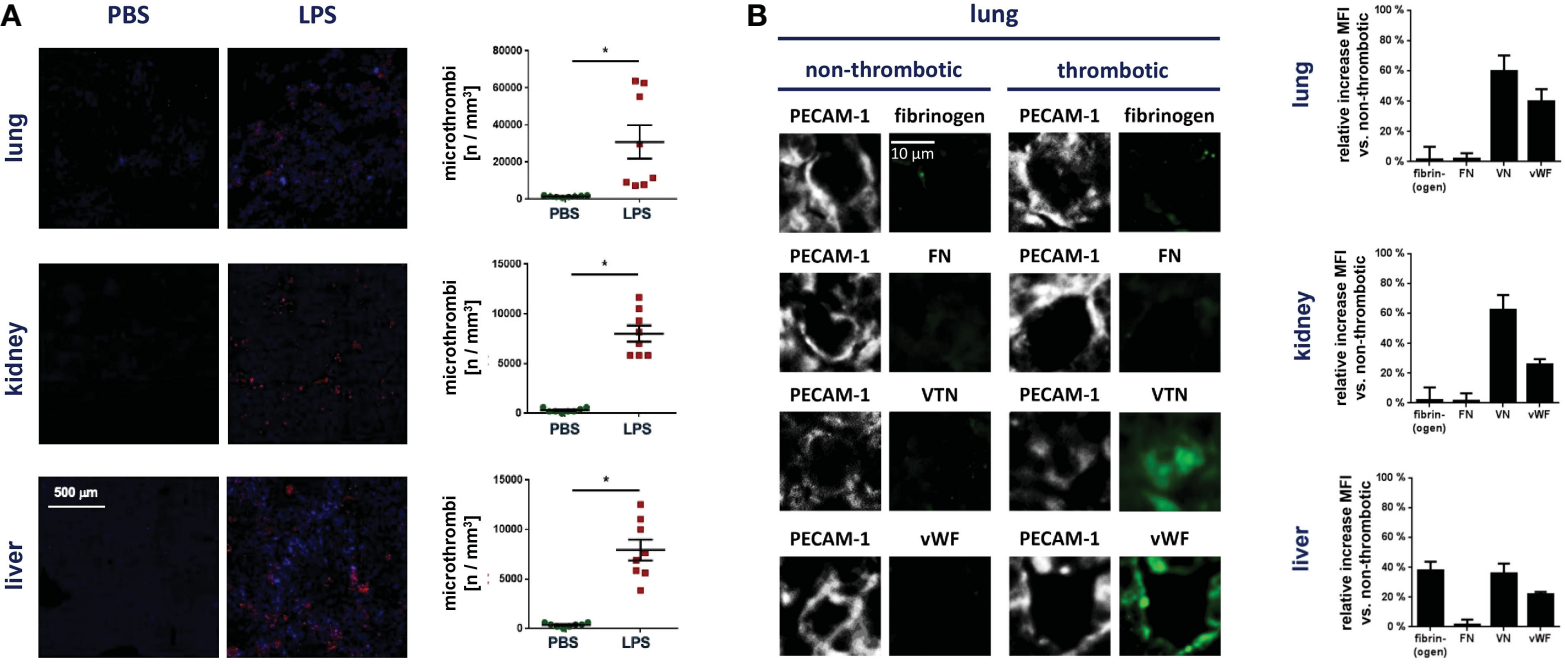
VN accumulates in the thrombotic microvasculature during systemic inflammation. **(A)** Accumulation of Gr-1^+^ immune cells (red) and GPIbβ^+^ platelets (blue) during thrombus formation in microvessels as analyzed by immunostaining and confocal microscopy in tissue sections of lungs, kidneys, and livers from WT mice treated with PBS or LPS, representative images and quantitative data for the occurrence of microthrombi are shown (mean ± SEM for n=7-20 per group; #p<0.05 vs. PBS). **(B)** The relative increase in the deposition of fibrin(ogen), FN, VN, or vWF in microvascular thrombi as compared to non-thrombotic microvessels of diseased animals was assessed by immunostaining and confocal microscopy, representative images are shown for lungs (PECAM-1/CD31 in gray; fibrin(ogen), FN, VN, or vWF in green) and quantitative data are shown for lungs, kidneys, and liver (mean ± SEM for n=4 per group).

**Figure 2:**
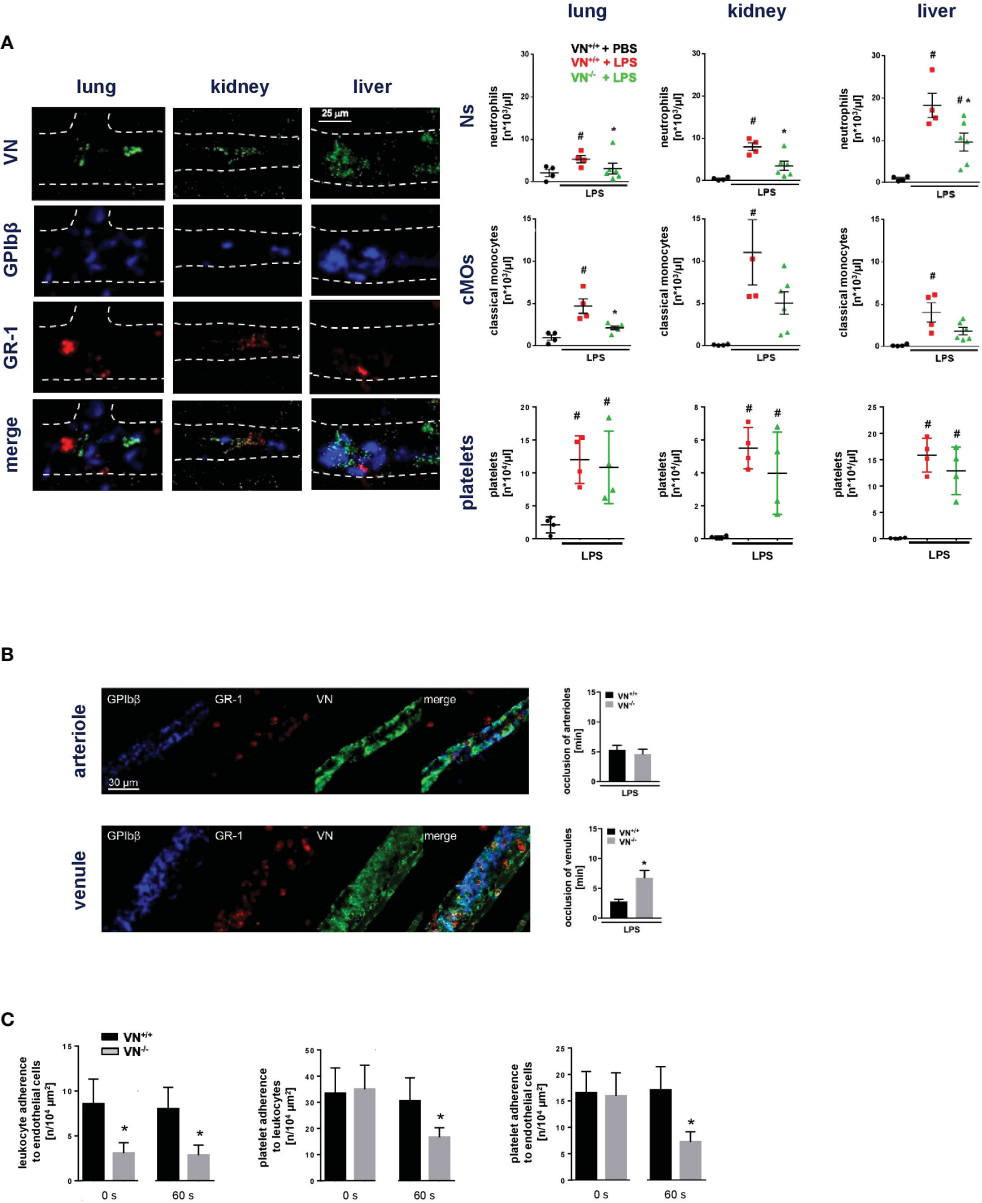
VN promotes microvascular immunothrombosis in systemic inflammation. **(A)**, left) Interactions of VN, GPIbβ^+^ platelets, and GR-1^+^ myeloid leukocytes in the hepatic, pulmonary, and renal microvasculature of WT mice treated with PBS or LPS as assessed by immunostaining and confocal microscopy, representative images are shown. **(A)**, right) Accumulation of neutrophils (Ns), classical monocytes (cMOs), and platelets (P) as assessed by flow cytometry in organ lysates from VN^+/+^ mice treated with PBS or LPS and VN^-/-^ mice treated with LPS, quantitative data are shown (mean ± SEM for n=4-6 per group; #p<0.05 vs. VN^+/+^+PBS; *p<0.05 vs. VN^+/+^+LPS). **(B)** Photochemical injury-elicited venular and arteriolar thrombus formation in the LPS-stimulated cremaster muscle of VN^+/+^ or VN^-/-^ mice as assessed by multi-channel *in vivo* microscopy, quantitative data for occlusion times as well as **(C)** for interactions of single platelets, myeloid leukocytes, and venular endothelial cells prior to induction of (0 s) or 60 s after induction of photochemical injury-elicited thrombosis are shown (mean ± SEM for n=4-6 per group; *p<0.05 vs. VN^+/+^).

In initial experiments, we sought to characterize the functional relevance of VN for microvascular immunothrombosis in systemic inflammation. Employing a mouse model of severe systemic inflammation, confocal laser scanning microscopy analyses of immunostained tissue samples detected various microvascular immunothrombi in liver, kidneys, and lungs of diseased animals, but not in healthy controls (**Figure 1A**). In these blood clots, significantly higher levels of VN and – to a lesser degree – of vWF were identified than in non-thrombotic microvessels (**Figure 1B**). Here, dense networks of VN tightly framed platelets and immune cells in the thrombotic microvasculature (**Figure 2A**). In contrast, fibrin(ogen) was only enriched in the thrombotic lower-shear liver sinusoids, but not in the thrombotic higher-shear pulmonary and renal microvessels, whereas FN did not preferentially accumulate in the thrombotic microvasculature of these organs (**Figure 1B**). Flow cytometry additionally revealed that numbers of neutrophils and – to a much lesser extent – numbers of classical monocytes and platelets in homogenates of inflamed tissues harvested from VN-deficient animals were lower than in wild-type controls (**Figure 2A**).

To directly evaluate the functional relevance of VN for microvascular clot formation, we used a model of photochemical injury-elicited microvascular thrombogenesis in the inflamed mouse cremaster muscle. Multi-channel *in vivo* microcopy demonstrated that thrombus formation in venules (but not in arterioles; **Figure 2B**) was significantly prolonged in VN-deficient mice as compared to WT controls. At baseline conditions prior to induction of thrombus formation, the number of neutrophils adherent to endothelial cells in postcapillary venules was significantly lower in VN-deficient animals than in WT controls, whereas VN-deficiency did not significantly alter adherence of platelets to endothelial cells or neutrophils. During thrombus formation, however, interactions between all these cell types were significantly reduced by VN-deficiency (**Figure 2C**). Moreover, flow chamber experiments revealed that VN – in contrast to fibrin(ogen) – facilitates platelet adhesion primarily under lower shear (**Figure S1**). Hence, our experimental data suggest that VN promotes microvascular immunothrombosis in inflamed tissue by supporting the multi-cellular interplay required for proper clot formation.

### GPIIb/IIIa controls platelet interactions in venular immunothrombosis

**Figure 3:**
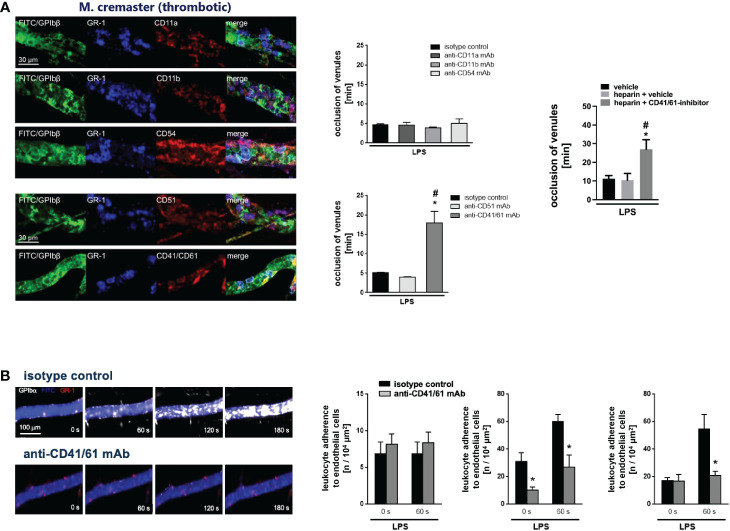
Microvascular immunothrombosis is controlled by the VN receptor CD41/CD61. **(A)** Presence of CD11a/LFA-1, CD11b/Mac-1, and CD54/ICAM-1, the VN interaction partners CD51 and CD41/CD61, as well as of GPIbβ^+^ platelets and GR-1^+^ myeloid leukocytes in venular immunothrombi as assessed by immunostaining and confocal microscopy in the LPS-stimulated cremaster muscle of WT mice, representative images are shown. Photochemical injury-elicited thrombus formation as assessed by multi-channel *in vivo* microscopy in LPS-stimulated cremasteric venules of WT mice receiving blocking monoclonal antibodies directed against CD11a, CD11b, CD54/ICAM-1, CD51, CD41/CD61, or heparin or heparin and a CD41/CD61 inhibitor, or isotype control antibodies/drug vehicle, representative data are shown (n=3-4 per group; #p<0.05 vs. isotype control). **(B)** Interactions of single GPIbβ^+^ platelets, GR-1^+^ myeloid leukocytes, and endothelial cells prior to induction of (0 s) or 60 s after induction of photochemical injury-elicited thrombosis in LPS-stimulated cremasteric venules of WT mice receiving isotype control or blocking monoclonal antibodies directed against CD41/CD61, representative images and quantitative data are shown (n=4 per group; *p<0.05 vs. isotype control).

Towards a more comprehensive understanding of VN-dependent venular thrombogenesis in inflamed tissue, we sought to define the role of different adhesion and signaling molecules in this process. The leukocyte β1 integrin VLA-4/CD49d and the β2 integrins LFA-1/CD11a and Mac-1/CD11b primarily mediate intravascular adherence of neutrophils to endothelial cells in inflamed tissue, whereas the integrins CD51/CD61 and CD41/CD61 (also: glycoprotein (GP) IIb/IIIa) serve as principal receptors of VN. Employing confocal laser scanning microscopy of immunostained cremasteric tissue whole mounts, all these molecules were detected in venular inflammatory thrombi. Interestingly, only antibody-mediated blockade of GPIIb/IIIa (but not blockade of the other molecules) significantly prolonged thrombus formation in inflamed postcapillary venules (**Figure 3A**), as evidenced in photochemical injury-elicited venular thrombogenesis. Whereas multi-channel *in vivo* microscopy further documented that blockade of GPIIb/IIIa significantly reduced interactions of platelets with endothelial cells or neutrophils during venular clot formation, neutrophil-endothelial cell interactions were not significantly altered (**Figure 3B**). Consequently, the VN receptor GPIIb/IIIa critically regulates platelet interactions in microvascular immunothrombosis.

### VN facilitates platelet aggregation *via* GPIIb/IIIa

**Figure 4:**
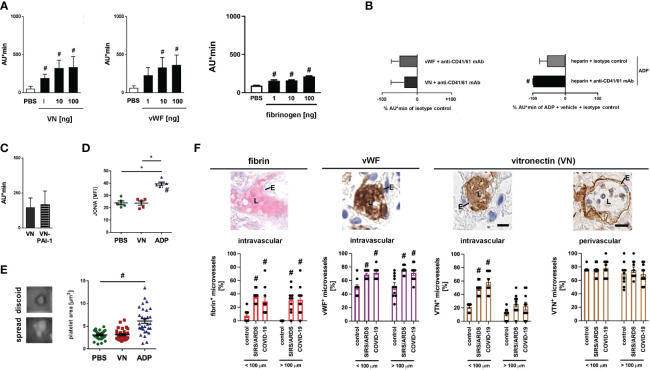
VN promotes platelet aggregation *via* CD41/61. **(A)** Aggregation of primary platelets from the peripheral blood of WT mice upon addition of different doses of recombinant mouse VN, vWF, or fibrinogen as assessed *in vitro* by rotational thrombelastometry, quantitative data are shown. Experiments were repeated upon addition of **(B)** isotype control antibodies or blocking monoclonal antibodies directed against CD41/CD61, upon stimulation with ADP after treatment with heparin and isotype control antibodies or blocking monoclonal antibodies directed against CD41/CD61, or **(C)** upon addition of VN-PAI-1 (mean ± SEM for n=4-6 per group; #p<0.05 vs. PBS). **(D)** Recognition of activated integrin CD41/CD61 by antibody JON/A on primary platelets from the peripheral blood of WT mice upon addition of PBS, VN, or ADP as assessed *in vitro* by flow cytometry (mean ± SEM for n=5 per group; #p<0.05 vs. PBS or VN). **(E)** Spreading on glass slides of primary platelets from the peripheral blood of WT mice upon addition of PBS, VN, or ADP as assessed *in vitro* by light microscopy, representative images and quantitative data are shown (mean ± SEM for n=22-30 per group; #p<0.05 vs. PBS). **(F)** Deposition of fibrin (bright red; upper left image), vWF (brown; upper left/middle image), and VN (brown; upper and right images) in the pulmonary microvasculature of patients with severe systemic infectious inflammation (COVID-19), severe systemic non-infectious inflammation (pancreatitis-associated SIRS/ARDS), and control patients as assessed by LADEWIG’s staining and immunohistochemistry (scale bars: 25 µm; L=lumen; E=endothelium). Representative images and quantitative data for pulmonary microvessels exhibiting inner diameters of more or less/equal than 100 µm are shown (mean ± SEM for n=10 patients).

In addition to VN and vWF, fibrin(ogen) is able to bind to GPIIb/IIIa. Application of heparin (which inhibits the generation of fibrin polymers) or defibrination with batroxobin (Defibrase^®^), however, did not significantly change the formation of immunothrombi in the inflamed cremaster muscle upon photochemical injury (**Figure 3A**; **Figure S2B**), thus corroborating the dominant role of VN and vWF in GPIIb/IIIa-dependent venular thrombogenesis. Employing rotational thrombelastometry, recombinant mouse VN dose-dependently increased the aggregation of primary mouse platelets, similar to vWF and fibrin(ogen) (**Figure 4A**). These effects of vWF and VN were severely compromised upon antibody blockade of their receptor GPIIb/IIIa (**Figure 4B**). In line with our *in vivo* results (**Figure 3A**), blockade of GPIIb/IIIa significantly reduced ADP-elicited platelet aggregation *in vitro* even after inhibition of fibrin formation by heparin, further highlighting the importance of the vWF/VN-GPIIb/IIIa axis for platelet aggregation. Application of VN-PAI-1 heteromers (which promote fibrinolysis), however, did not significantly alter platelet aggregation (**Figure 4C**). Moreover, VN did not directly activate platelets as indicated by unchanged surface expression of GPIIb/IIIa in high affinity conformation in flow cytometry (**Figure 4D**) and did not induce the spreading of these cellular blood components (**Figure 4E**; **Figure S2A**). Collectively, VN primarily mediates the aggregation of platelets by involving its receptor GPIIb/IIIa.

### VN accumulates in pulmonary microvessels of patients with severe systemic non-infectious and infectious inflammatory pathologies

Immunothrombotic dysregulation is a key element in the pathogenesis of severe non-infectious and infectious systemic inflammatory pathologies including COVID-19 (36). Mechanistically, SARS-CoV-2 is thought to bind to its endothelial receptor angiotensin converting enzyme-2 (ACE2) which induces a pro-inflammatory and pro-coagulative phenotype in the infected host cells (37, 38). Microvascular thrombi are likewise found in lungs of patients with severe cases of non-infectious pancreatitis-associated SIRS/ARDS promoting organ tissue damage and functional failure. Here, the pancreatitis-induced systemic inflammatory response promotes direct (*e.g.*, complement-based ‘membrane attack complex’) as well as indirect (*e.g.*, elevated intravascular levels of pro-inflammatory cytokines) injury to the microvascular endothelium, ultimately resulting in a pro-inflammatory and pro-coagulative phenotype (39). These processes lead to a massive accumulation of immune cells and platelets in the affected microvasculature, eventually resulting in life-threatening organ dysfunction and failure (40–42). As a translational perspective of our study, we therefore sought to evaluate the prevalence of VN in the lung vasculature of patients with severe non-infectious (pancreatitis-associated SIRS/ARDS) and infectious (COVID-19-associated) systemic inflammation. Immunohistochemical analyses of autopsy specimens detected a massive accumulation of VN intravascularly – but not in the underlying subendothelial matrix – particularly in microvessels of the pulmonary vasculature of COVID-19 and pancreatitis-associated SIRS/ARDS patients. Conversely, deposition of fibrin(ogen) and vWF was found to be elevated both in micro- and macrovessels and was additionally less pronounced in microvessels as compared to VN under systemic inflammatory conditions (**Figure 4F**). Noteworthy, microvascular pulmonary VN deposition was slightly lower in pancreatitis-associated SIRS/ARDS patients as compared to COVID-19 patients.

## Discussion

To decipher the functional role of VN for microvascular immunothrombosis in systemic inflammation, we employed an animal model of severe systemic inflammation. Expectedly, we found multiple immunothrombi in the hepatic, renal, and pulmonary microvasculature of the diseased animals. In these blood clots, dense networks of VN and – to a lesser degree – of vWF encased platelets and immune cells. Of note, fibrin(ogen) was only enriched in the thrombotic lower-shear liver sinusoids, but not in the thrombotic higher-shear microvessels of inflamed lungs and kidneys, whereas FN did not preferentially accumulate in the thrombotic microvasculature of these organs. With respect to these distinct deposition patterns of VN in inflammatory microvascular clots, we hypothesized that this matricellular protein is critical for microvascular thrombogenesis in systemic inflammation. In line with this assumption, we found that VN promotes the accumulation of neutrophils and – only slightly – of classical monocytes, but not of platelets, in inflamed tissues. Mechanistically, we have recently demonstrated that VN stabilizes the adhesion of neutrophils, but not of classical monocytes or platelets, to the inflamed microvascular endothelium by coordinating proper β2 integrin clustering on the surface of these immune cells (43). Since neutrophils are well-known to facilitate the trafficking of classical monocytes under inflammatory conditions (18, 44–47), the reduced microvascular accumulation of these immune cells in VN-deficient animals might be the consequence of diminished neutrophil responses. Importantly, these analyses included both platelets firmly adherent to non-thrombotic microvessels (for which VN is not relevant) (43) and platelets forming microvascular clots. In a more detailed analysis, we here show that VN directly promotes thrombus formation in venules by supporting the intravascular interplay of platelets with endothelial cells or already adherent leukocytes. Since interactions of blood cells and the microvascular endothelium are strictly dependent on shear (48), these divergent effects of VN on immune cell and platelet interactions might be explained by the different rheological conditions present in different vascular beds. Specifically, pertubations arising from intravascularly adherent leukocytes during inflammatory clot formation in high-shear postcapillary venules have recently been shown to shape the rheological environment for platelet accumulation and thereby promote microvascular thrombogenesis (18). Moreover, we identified VN to facilitate platelet adhesion only under lower shear, further indicating that this matricellular protein is particularly relevant for venular platelet accumulation at later stages of thrombogenesis when shear progressively decreases in clotting microvessels.

Intravascular adherence of leukocytes to the microvascular endothelium is mediated by interactions of the leukocyte β1 integrin VLA-4/CD49d and the β2 integrins LFA-1/CD11a and Mac-1/CD11b with endothelial interaction partners of the immunoglobulin superfamily (*e.g.*, ICAM-1/CD54) (49, 50). In the present study, we found that these molecules are present in inflammatory microvascular thrombi, but are not functionally relevant for microvascular immunothrombosis in inflamed tissue. These data are in line with our previous observations documenting that leukocyte recruitment to forming microvascular clots is not critical for inflammatory thrombogenesis (18).

The VN receptor integrin CD51/CD61 (αVβ3) and GPIIb/IIIa (αIIbβ3) on leukocytes or platelets are the principal cellular interaction partners of VN in the microvasculature. Accordingly, we here detected both molecules in microvascular thrombi, but show that only GPIIb/IIIa contributes to thrombus formation in inflamed postcapillary venules. Similar to VN, we found that this glycoprotein regulates platelet-neutrophil and platelet-endothelial cell interactions, but does not affect endothelial interactions of already intravascularly adherent neutrophils. Hence, our data suggest that the VN-GPIIb/IIIa axis critically participates in immunothrombosis of the venular microvasculature by orchestrating a multi-cellular platelet interplay required for proper clot formation.

Upon platelet activation, the integrin complex GPIIb/IIIa promotes adhesion and aggregation of these cellular blood components by sequential interactions with deposited vWF and, subsequently, with fibrinogen. Importantly, however, fibrin(ogen) is barely enriched in the inflamed high-shear microvasculature and has been shown to be largely dispensible for inflammatory thrombus formation in the arteriolar and venular microvessels (18). These observations are additionally corroborated by the lacking effect of heparin (interfering with the plasmatic coagulation) on venular thrombogenesis in systemic inflammation observed in our study. Instead, we show that VN exhibits potent pro-aggregatory effects on platelets, similar to vWF which were largely dependent on their common receptor GPIIb/IIIa. Consequently, VN might be able to substitute for fibrin(ogen) in platelet aggregation during immunothrombosis in vessels with low fibrin(ogen) deposition (51), whereas in the hepatic microvasculature (exhibiting strong deposition of fibrin(ogen)) fibrin(ogen) is required for thrombus formation (6).

Importantly, binding of VN to its interaction partner PAI-1 increases the inhibitory potential of this protease inhibitor to compromise plasmin-dependent fibrin degradation elicited by urokinase-type or tissue plasminogen activator in fibrinolysis. In accordance with the previously observed dispensability of fibrin(ogen) in microvascular immunothrombosis, however, heteromerization of VN and PAI-1 did not significantly alter platelet aggregation in our experiments. Similarly, heteromerization of VN and PAI-1 did not enhance the pro-thrombotic potential of the single proteins (52) and PAI-1 deficiency did not alter microvascular clot formation (53) in previous studies. Furthermore, we found that VN neither directly activates platelets nor induces the spreading of these cellular blood components. Collectively, this indicates that VN promotes platelet aggregation in immunothrombosis of the venular microvasculature by cross-linking activated platelets through GPIIb/IIIa.

Early reports from Wuhan (China) already demonstrated that abnormal coagulation parameters such as elevated blood levels of fibrin degradation products are associated with impaired survival in severe cases of COVID-19 (54–57). While VN is released by the liver and circulating platelets, the intra- and perivascular deposition of this matricellular protein determines its functional presence in the circulation. In this context, we identified a massive accumulation of VN in the pulmonary microvasculature of patients with severe systemic infectious inflammation (COVID-19) as well as of patients with severe systemic non-infectious inflammation (pancreatitis-associated SIRS/ARDS). In contrast, fibrin(ogen) and vWF deposition in lungs of COVID-19- and pancreatitis-associated SIRS/ARDS-patients was elevated in micro- and macrovessels while it was less pronounced in the microvasculature as compared to VN. Although experimental systemic inflammation induced by LPS in mice is not equivalent to systemic inflammation mediated by SARS-CoV-2 progression or acute pancreatitis in humans, our observations collectively indicate that VN might participate in microvascular immunothrombotic dysregulation under severe systemic inflammatory conditions. In this context it is interesting that first clinical data on anti-platelet treatment using a GPIIb/IIIa antagonist (tirofiban) or other anti-platelet agents including a COX inhibitor (aspirin) or ADP receptor antagonist (clopidogrel) showed protective effects in severe COVID-19 (58, 59). Noteworthy, treatment with these anti-platelet compounds also attenuated the risk of developing acute respiratory distress syndrome in non-COVID-19 patients, but did not reduce their mortality (60, 61), pointing to disease-specific effects of these therapeutic interventions. Consequently, blocking the VN–GPIIb/IIIa axis might be particularly beneficial in severe systemic inflammatory pathologies such as COVID-19.

In conclusion, our data show that VN accumulates in the microvasculature during systemic inflammation, where this matricellular protein establishes a dense molecular network. In this manner, it supports interactions of platelets, immune cells, and endothelial cells through the platelet surface receptor GPIIb/IIIa, thus effectively promoting immunothrombosis. Targeting the VN-GPIIb/IIIa axis with readily available GPIIb/IIIa inhibitors might therefore represent a promising strategy to counteract microvascular immunothrombotic dysregulation in severe systemic inflammation.

## Data availability statement

The raw data supporting the conclusions of this article will be made available by the authors, without undue reservation.

## Ethics statement

The studies involving human participants were reviewed and approved by Ethikkommission der Technischen Universität München Ismaninger Straße 22 81675 München. Written informed consent for participation was not required for this study in accordance with the national legislation and the institutional requirements. The animal study was reviewed and approved by Regierung von Oberbayern Sachgebiet 55.2 - Rechtsfragen Gesundheit, Verbraucherschutz und Pharmazie Maximilianstraße 39 80538 München.

## Author contributions

BU, GZ, and CR contributed to conception and design of the study. BU and CR wrote the first draft of the manuscript. BU, FH, JSH, JL, VS, JH, ZW, KSte, BS, AB, OK, JCH, BW, MC, TL, KSto, KSta, TB, MM, CS, WW, GZ, and CR contributed to acquisition, analysis, and/or interpretation of data for the work as well as revised and/or wrote sections of the manuscript. All authors contributed to manuscript revision, read, and approved the submitted version.

## Supplementary material

Supplementary Figure 1 Platelet adhesion under flow. Platelet adhesion under flow exposed to varying shear stress as assessed in flow chambers coated with VN or fibrin(ogen) and perfused with mouse blood, quantitative data (mean ± SEM for n=4 – 6 per group; #p<0.05 vs. 1 dyne/cm²) and representative images are shown.

Supplementary Figure 2 Effect of VN on platelet spreading and effect of defribrination on venular thrombosis. **(A)** Spreading on glass slides coated with collagen I or fibrin(ogen) of primary platelets isolated from the peripheral blood of WT mice upon addition of PBS, VN, or ADP as assessed *in vitro* by light microscopy, representative images and quantitative data are shown (mean ± SEM for n=25 per group; #p<0.05 vs. PBS). **(B)** Photochemical injury-elicited venular thrombus formation in the LPS-stimulated cremaster muscle of WT mice as assessed by multi-channel *in vivo* microscopy, quantitative data for venular occlusion times after induction of photochemical injury-elicited thrombosis are shown (mean ± SEM for n=4 per group; *p<0.05 vs. vehicle).
