# Supplementary figures and images for: Vitronectin promotes immunothrombotic dysregulation in the venular microvasculature
Source: Front Immunol. 2023 Feb 8;14:1078005. doi: 10.3389/fimmu.2023.1078005 (PMC9945350; doi:10.3389/fimmu.2023.1078005)

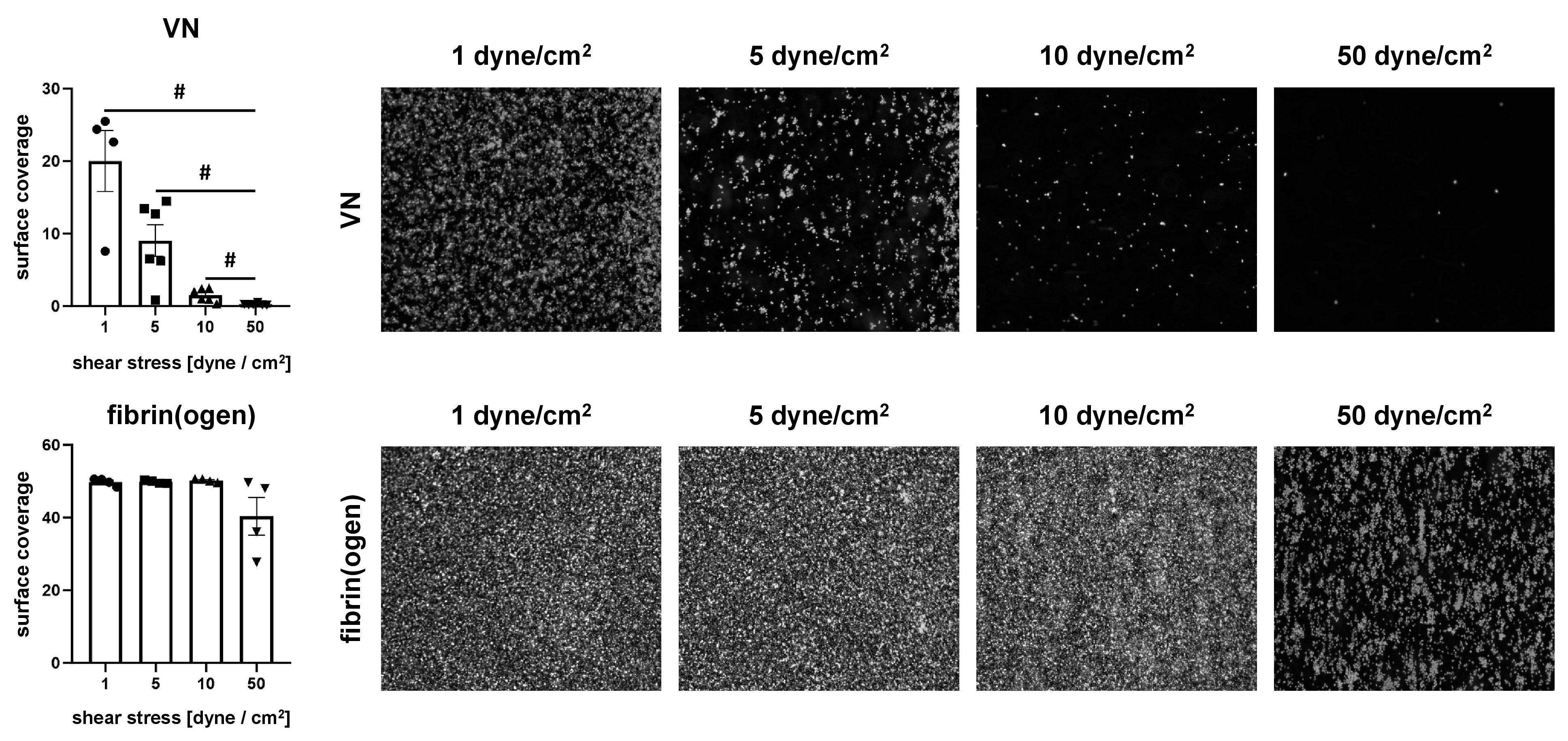

Supplement: Supplementary Figure 1 — Platelet adhesion under flow. Platelet adhesion under flow exposed to varying shear stress as assessed in flow chambers coated with VN or fibrin(ogen) and perfused with mouse blood, quantitative data (mean ± SEM for n=4 – 6 per group; #p<0.05 vs. 1 dyne/cm²) and representative images are shown. [file Image_1.jpeg]

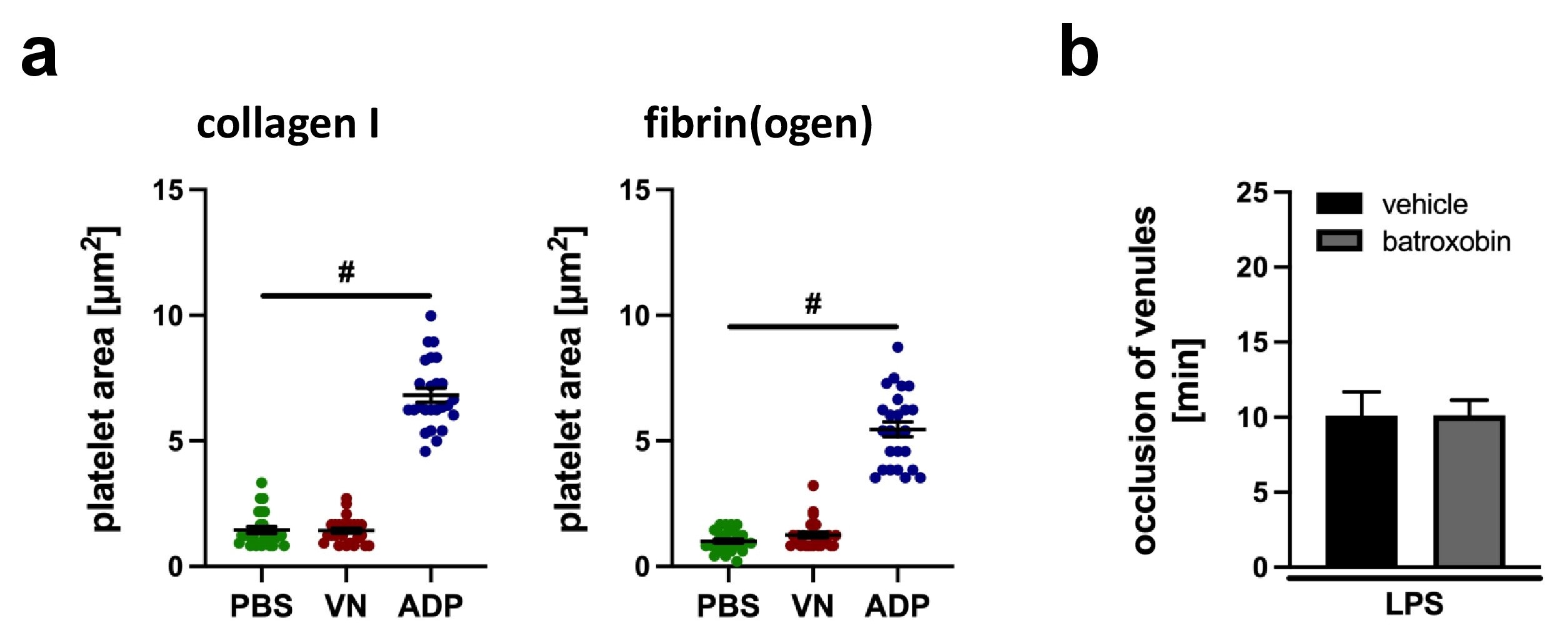

Supplement: Supplementary Figure 2 — Effect of VN on platelet spreading and effect of defribrination on venular thrombosis. (A) Spreading on glass slides coated with collagen I or fibrin(ogen) of primary platelets isolated from the peripheral blood of WT mice upon addition of PBS, VN, or ADP as assessed in vitro by light microscopy, representative images and quantitative data are shown (mean ± SEM for n=25 per group; #p<0.05 vs. PBS). (B) Photochemical injury-elicited venular thrombus formation in the LPS-stimulated cremaster muscle of WT mice as assessed by multi-channel in vivo microscopy, quantitative data for venular occlusion times after induction of photochemical injury-elicited thrombosis are shown (mean ± SEM for n=4 per group; *p<0.05 vs. vehicle). [file Image_2.jpeg]
